# Labour market participation after spinal cord injury. A register-based cohort study

**DOI:** 10.1038/s41393-023-00876-4

**Published:** 2023-01-30

**Authors:** Annette Halvorsen, Aslak Steinsbekk, Annelie Schedin Leiulfsrud, Marcel W. M. Post, Fin Biering-Sørensen, Kristine Pape

**Affiliations:** 1 Clinic of Physical Medicine and Rehabilitation, Department of Spinal Cord Injuries, St. Olavs hospital, Trondheim University Hospital, Trondheim, Norway; 2 Department of Medical Quality Registries, St. Olavs hospital, Trondheim University Hospital, Trondheim, Norway; 3 Department of Public Health and Nursing, Norwegian University of Science and Technology, Trondheim, Norway; 4 Department of Neuro Medicine and Movement Science, Faculty of Medicine and Health Sciences, NTNU-Norwegian University of Science and Technology, Trondheim, Norway; 5 Center of Excellence for Rehabilitation Medicine, UMC Utrecht Brain Centre, University Medical Centre Utrecht, University Utrecht and De Hoogstraat Rehabilitation, Utrecht, The Netherlands; 6 University of Groningen, University Medical Centre Groningen, Centre for Rehabilitation, Groningen, The Netherlands; 7 Section for Spinal Cord Injuries, Department for Brain and Spinal Cord Injuries, Copenhagen University Hospital, Rigshospitalet, and Department for Clinical Medicine, University of Copenhagen, Copenhagen, Denmark

**Keywords:** Epidemiology, Spinal cord diseases

## Abstract

**Study design:**

A register based cohort study.

**Objectives:**

To investigate labour market participation following spinal cord injury (SCI) and to describe the impact of personal and SCI characteristics.

**Setting:**

Norway.

**Methods:**

Persons registered with SCI in the Norwegian SCI registry 2011–2017, and matched reference individuals without SCI from the general population (named controls) were followed for up to six years after injury using national registry data on employment, education, income, and social security benefits. Main measures of labour market participation were: (1) Receiving any amount of pay for work, and (2) Receiving sickness and disability benefits.

**Results:**

Among the 451 persons with SCI (aged 16–66 years and working before injury), the estimated percentages receiving pay for work and sickness and disability benefits in the sixth years after injury were 63% (95% CI 57–69) and 67% (95% CI 61–72).

Corresponding percentages for the controls (*n* = 1791) were 91% (95% CI 90–93) for receiving pay for work and 13% (95% CI 12–15) for receiving sickness and disability benefits. Among persons with SCI, less severe neurological outcome, higher level of education, younger age at injury, and a stronger pre-injury attachment to employment (higher employment income, having an employer, less receipt of benefits), were associated with higher labour market participation.

**Conclusion:**

SCI substantially decreased labour market participation up to six years after injury compared to matched controls. Even if a relatively large proportion of persons with SCI remained in some degree of work activity, more than half did so in combination with receiving benefits.

## Introduction

Employment is a key rehabilitation outcome for people with spinal cord injury (SCI), and it tends to be positively associated with adjustment to SCI, life satisfaction, a sense of purpose, mental stimulation, social contact and well-being [1]. The level of employment among people with SCI is positively influenced by a number of factors, such as personal factors (younger age at time of injury, higher level of education, higher motivation), SCI-related characteristics (less severe neurological outcome) and employment-related factors (support from the employer, possibility to continue working in the same organisation) [2–6].

Most people with SCI can potentially be in employment if they get access to appropriate work accommodations [1]. However, the average employment rate among 9875 persons with SCI in 22 countries across the world was 38% (ranging from 10% to 61%), which was considerably lower than in the respective general working populations [7]. Contextual country-level factors, such as labour market systems and policies with respect to social security, vocational rehabilitation and employment, may explain some of the differences in employment levels across countries [8].

Norway has a well-developed welfare and health care system with universal rights to health and welfare provisions, a strong policy emphasis on high employment, and high expenditure on return to employment measures after sickness or injury. Compared with other European countries, a large portion of the Norwegian population is on sickness and disability benefits [9]; 17% in the 18–66 age group [10]. Still, in the last quartile of 2021, 72.3% of the population aged 15–74 was part of the workforce [11]. The proportion employed following SCI in Norway have been reported to range from 35% to 52% in different studies [4, 5, 7, 12], which shows that there is considerable employment gap between persons with SCI and the general population.

To gain further knowledge regarding labour market participation in the SCI population, there is a need for longitudinal studies with information on both employment and sickness and disability benefits, to achieve a more complete picture [13]. Norway is an ideal place to perform such studies due to its national SCI registry which can be linked to several population-based databases [14].

The overall objective was to investigate labour market participation up to six years following SCI. The specific aims were: (1) to describe labour market participation post-SCI for persons receiving pay for work (>0 Norwegian Kroner (NOK)/Euro (EUR)) the year before SCI; (2) to compare labour market participation following SCI with a matched group from the general population; (3) to describe the impact of personal and SCI characteristics on labour market participation after SCI.

## Methods

### Study design

Cohort study following persons with SCI from the Norwegian SCI registry (NorSCIR), and a matched reference group without SCI taken from the general Norwegian population (named “controls”), for one year before injury and up to six years after injury, using national registry data on employment, education, income, and social security benefits.

NorSCIR is a national medical quality registry for SCI care. All patients with traumatic or non-traumatic SCI admitted for first rehabilitation to one of the three Norwegian specialized SCI departments and who give their consent are included in the registry. Annual reviews show that this registry covers > 90% of the incidence population [15].

Ethical approval was obtained from the Regional Committee for Medical and Health Research Ethics in Central Norway (2018/294/REK-midt). Registration in the NorSCIR is voluntary with a written informed consent before registration occurs. Persons giving consent to participate in NorSCIR accept that their information can be used for research purposes, including linkage with a range of national registers.

### Study samples

Included in the SCI sample were all persons who experienced traumatic or non-traumatic SCI, were admitted to one of the three SCI units in Norway and registered in the NorSCIR from 01.01.2011 to 31.12.2017, were in working age (16 to 66 years) at time of injury, living in Norway in the month before injury, and “eligible” for work by receiving any pay for work (>0 NOK/EUR) in the year before SCI. We used 66 years of age as the upper limit because the legal retirement age is 67 years in Norway. The lower limit of 16 years was chosen as this is the last year of compulsory education.

Matched reference individuals from the general population (matching the SCI sample for year of birth, sex, county of residence, and level of education) were randomly drawn from population registries by Statistics Norway. Participants in this sample are in this study named “controls”. Five controls were drawn for each patient. Only controls living in Norway and employed, according to the same definition as for the SCI sample, were included in the analyses.

### Data sources

NorSCIR [16] provided information on personal and injury characteristics: Age at injury, sex, date of acute hospital admission, neurological classification and cause of injury.

Statistics Norway (SSB) [17, 18] provided data on income from work, registration status (dates of death and emigration), highest attained educational level, ongoing education, year of birth, county of residence, and sex (controls).

The Norwegian Labour and welfare administration (NAV) [19, 20] provided information on dates of sickness absence benefit, work assessment allowance, disability pension, old age pension, and employment status.

### The Norwegian social security system

All persons who are either residents or working as employees in Norway are insured under the National Insurance Scheme, managed by NAV [19]. Employed people can be granted sick leave compensation covering up to 100% of income for a period of maximum 52 weeks if they are unable to work due to an illness or injury. After 52 weeks, employees with a reduction in work ability of at least 50% due to illness or injury may apply for long-term benefits (work assessment allowance (AAP) or disability pension (DP)) to compensate for loss of income. While AAP is a temporary benefit (max 3 years) requiring active treatment and/or rehabilitation measures, DP is granted on a permanent basis to those whose earning capacity is permanently reduced. The total allowance from AAP and DP is approximately 66% of the income from the three best payed of the last five years before disability and up to maximum six times the National insurance basic amount (G) for each year (1G = 106 399 Norwegian kroners, approximately 11 033 euro (as of 21.3.2022)).

### Linkage

The SCI and control sample were linked to the various registry data by an identification key created by Statistics Norway using the unique 11-digit personal identity number given to all Norwegian citizens.

### Follow-up period

The start of the follow-up period was 12 months before the date of injury. The date of injury was set to the date of acute hospital admission registered in NorSCIR for the SCI sample, and controls were assigned the same date as their respective matched persons with SCI. Participants were censored at the date/month of emigration, death, 67 years’ birthday, last available data (31.12.2020) or month 72 after injury, whichever came first. The choice of ending follow-up at 72 months/6 years post-SCI was made since less than half of the cohort (only those injured 2011-2013) could be followed past this point. The total follow-up period was divided into a maximum of seven 1-year time intervals (−1 (year before injury), 0–1, 1–2, 2–3, 3–4, 4–5, 5–6 (years after injury)).

### Measures of labour market participation post-spinal cord injury

Main measures of labour market participation were: (1) Receiving any amount of pay for work (>0 NOK/EUR), and (2) Receiving sickness and disability benefits, which included sickness absence benefit, work assessment allowance and/or disability pension, to compensate for loss of income. Additional outcomes included: (1) Mean employment income as a continuous variable and (2) Each of the sickness/disability benefits separately (Table 1).

**Table 1:** Description of measures of labour market participation.

Main outcomes	Data source	Variable(s)	Construction	Assessment periods
Receiving any amount of pay for work	SSB(17, 18)	Any income from work and self-employment. (Annual observations)	Receiving pay for work (pay for work>0 NOK/EUR) (1) or not receiving pay for work (no pay for work) (0).	1-year intervals from date of injury to six years (72 months) after injury.
Receiving sickness and disability benefits	NAV(19,20)	1. Sickness absence benefits. 2. Work assessment allowance. 3. Disability pension. (Monthly observations)	Receiving any of the three benefits (1) or not receiving any benefit (0).*	1-year intervals from one year (12 months) before injury to six years (72 months) after injury.
**Additional outcomes**	**Data source**	**Variable(s)**	**Construction**	**Assessment periods**
Employment income	SSB(17, 18)	Income from work and self-employment. (Annual observations)	Level of employment income (in NOK(EUR)) as continuous variable	1-year intervals from one year before injury to six years (72 months) after injury.
Receiving sickness absence benefit	NAV(19,20)	Sickness absence benefit. (Monthly observations)	Receiving the benefit (1) or not receiving the benefit (0).*	1-year intervals from one year (12 months) before injury to six years (72 months) after injury.
Receiving work assessment allowance	NAV(19,20)	Work assessment allowance. (Monthly observations)	Receiving the benefit (1) or not receiving the benefit (0).*	1-year intervals from one year (12 months) before injury to six years (72 months) after injury.
Receiving disability pension	NAV(19,20)	Disability pension. (Monthly observations)	Receiving the benefit (1) or not receiving the benefit (0).*	1-year intervals from one year (12 months) before injury to six years (72 months) after injury.

^*^The monthly observations were used as the basis for outcome assessment in 1-year intervals. To be registered with outcome/benefit, the persons had to be registered with benefits in at least 9 out of 12 months (or at least 75% of monthly registrations during the 1-year interval).

*SSB* Statistics Norway, *NAV* The Norwegian Labour and Welfare Administration, *NOK* Norwegian kroner, *EUR* Euro.

### Study variables

Study variables available for the SCI sample (from NorSCIR) included sex, age at injury (16–29, 30–39, 40–49, 50–59, 60–66 years), date of acute hospital admission for SCI (2011–2014, 2015–2017), cause of injury (traumatic, non-traumatic), and neurological status.Categorisation of neurological status was done using the International Standards for Neurological Classification of SCI [21], including neurological level of injury and American Spinal Injury Association Impairment Scale (AIS) grade, to create four SCI impairments groups (level and AIS): Tetraplegia (C1-C8) AIS A, B or C; Tetraplegia (C1-C8) AIS D, Paraplegia (T1-S5) AIS A, B or C; Paraplegia (T1-S5) AIS D, E. In cases of missing neurological status at discharge, this was replaced with the classification at admission. Those with AIS E at discharge had neurological level at T1 or lower prior to the last examination and were categorized into group Paraplegia AIS D, E.

Baseline variables for both the SCI and control samples were assessed during one year before injury (from SSB and NAV) and included highest educational level (primary, secondary, higher education), ongoing education, employment income (NOK 0–299.999, 300.000–499.999, 500.000–999.999, and ≥1.000.000), employment status (having a registered employer for at least 1 month in the year before injury or not) and receipt of sickness or disability benefits (receiving benefits in at least one month in the year before injury or not).

### Statistical analysis

Characteristics of the patient and control samples were presented with descriptive statistics.

We explored the association between time during follow-up and labour market participation using general estimation equations’ (GEE) logistic regression analyses. Analyses included time as year in relation to injury (each year as a category, from year before to sixth years after) and repeated measures of the two dichotomous labour market participation outcomes (pay for work and sickness/disability benefits, assessed each year of follow-up for each participant). We performed separate analyses for the SCI and control samples, with adjustment for age, sex, and educational level. Estimates from the analyses were used to calculate and graphically present the level (percentage) of labour market participation at each 1-year interval during follow-up.

For the SCI sample, we used a similar approach to assess labour market participation over time for subgroups of age, sex, level of education, SCI impairment, and cause of injury. A separate GEE analysis was performed for each grouping variable by including it in the GEE model and adding an interaction term with the time variable. All analyses were adjusted for age, sex, and level of education.

For persons with SCI, we further explored the impact of various personal and SCI characteristics on labour market participation after injury using three different models (GEE logistic regression, with six repeated outcome assessments; from injury to six years after). Model A included adjustment for time, age group, sex, and level of education, Model B included additional adjustment for injury variables (SCI impairment group, cause of injury, year of injury), and Model C further added adjustment for pre-injury employment (employment income, employment status and medical benefits before injury).

We compared labour market participation between persons with SCI and controls within matched groups using fixed-effect logistic regression models. This analysis compares persons with SCI only with their designated controls, automatically adjusting for year of birth, sex, county of residence, and level of education (matching variables), and also accounting for the unequal number of controls per patient in the total samples. Estimates from the analyses were used to calculate the absolute and relative difference (prevalence difference in %-points and odds ratio with 95%CI) between the SCI and control sample for labour market participation at one year before injury, 1–3 years after injury and 4–6 years after injury.

Supplementary analyses included alternative outcome measures for labour market participation (assessed each year of follow-up for each participant); (1) Mean employment income as a continuous variable (SCI and control samples) and (2) each of the sickness/disability benefits separately; sickness absence benefit, AAP and DP (SCI sample only). The association between time (year in relation to injury) and each outcome was analysed using GEE linear or logistic regression, with an adjustment for age, sex and education.

Stata® version 16.0 (StataCorp, College Station, Texas, USA) was used for all statistical analyses.

## Results

Of the 751 persons registered in the NorSCIR from 01.01.2011 to 31.12.2017, 300 persons were excluded (aged ≥ 67 (*N* = 179), pay for work = 0 (*N* = 113, of which 75 were on full-time medical benefits), not living in Norway (*N* = 8)). Thus, 451 participants with SCI were included in the SCI sample.

After excluding controls aged ≥ 67, not living in Norway, and with pay for work = 0 (same criteria as for the SCI sample), 1791 persons matched to 443 persons with SCI remained in the control sample. In the SCI sample, 8 persons had no controls, 12 persons had one control, 33 persons had two controls, 68 persons had three controls, 141 persons had four controls and 189 persons had five controls.

The SCI and control samples were quite similar regarding age, sex, and education (Table 2). Controls had a slightly higher annual employment income and received less sickness and disability benefits during the year before SCI compared with the persons with SCI.

**Table 2:** Descriptive characteristics of the patients with spinal cord injury (*N* = 451) and matched controls (*N* = 1791). *N* (%) or mean (sd).

Characteristics	SCI population	Control population
	*N* = 451	*N* = 1791
***Mean age at injury, years (sd)***	44.0 (14.8)	43.3 (14.4)
***Age groups at injury N (%)***		
16–29 years	102 (22.6)	422 (23.6)
30–39 years	68 (15.1)	281 (15.7)
40–49 years	90 (20.0)	375 (20.9)
50–59 years	111 (24.6)	431 (24.1)
60–66 years	80 (17.7)	282 (15.8)
***Sex N (%)***		
Male	343 (76.1)	1374 (76.7)
Female	108 (24.0)	417 (23.3)
***Level of education year before injury N (%)***		
Primary	117 (25.9)	395 (22.1)
Secondary	210 (46.6)	893 (49.9)
Higher	124 (27.5)	503 (28.1)
***Ongoing education year before injury N (%)***		
Yes	34 (7.5)	173 (9.7)
No	417 (92.5)	1618 (90.3)
Having an employer year before injury (at least 75% *)		
Yes	335 (74.3)	1373 (76.7)
No	116 (25.7)	418 (23.3)
***Having an employer at least one moth in year before injury***		
Yes	386 (85.6)	1545 (86.3)
No	65 (14.4)	246 (13.7)
***Mean annual employment income before SCI in NOK (sd)***	409.859 (316.792)	438.341 (387.143)
***Mean annual employment income before SCI in EUR (sd)***	42.540 (32.880)	45.496 (40.182)
***Employment income groups N (%)***		
0–299.999 NOK (0-31.137 EUR)	171 (37.9)	598 (33.6)
300.000–499.999 NOK (31.138-51.896 EUR)	130 (28.8)	589 (33.1)
500.000 – 999.999 NOK (51.897-103.791 EUR)	129 (28.6)	536 (30.1)
≥1.000.000 NOK (>103.792)	21 (4.7)	57 (3.2)
Receiving any sickness or disability benefit year before injury (at least 75%**)		
Yes	77 (17.1)	154 (8.6)
No	374 (82.9)	1637 (91.4)
***Receiving any sickness or disability benefits in at least one month in year before injury***		
Yes	164 (36.4)	415 (23.2)
No	287 (63.6)	1376 (76.8)
***SCI Characteristics***		
***Cause of injury N (%)***		
Traumatic	301 (66.7)	
Non-traumatic	150 (33.3)	
***Impairment groups (Level and AIS) N (%)***		
Paraplegia, AIS D-E	159 (35.3)	
Tetraplegia, AIS D	113 (25.1)	
Paraplegia, AIS A-C	109 (24.2)	
Tetraplegia, AIS A-C	58 (12.9)	
Unknown or not applicable	12 (2.7)	
***Year of injury N (%)***		
2011	54 (12.0)	
2012	58 (12.9)	
2013	61 (13.5)	
2014	73 (16.2)	
2015	82 (18.2)	
2016	60 (13.3)	
2017	63 (14.0)	

*Being registered with employer in at least 9 out of 12 months (or at least 75% of monthly registrations during the 1-year interval).

**Being registered with sickness and disability benefits in at least 9 out of 12 months (or at least 75% of monthly registrations during the 1-year interval).

*NOK* Norwegian kroner, *EUR* Euro, *SCI* spinal cord injury, *AIS* American Spinal Injury Association Impairment Scale.

The median follow-up time for the SCI sample was 57 months.

The median follow-up time for the patient sample was 57 months (lower quartile 38, and upper quartile 72), and 58 months for the controls (lower quartile 42, and upper quartile 72) (data not presented). The number (and %) of participants receiving pay for work and/or sickness and disability benefits each year of follow-up is provided for both samples in supplemental Table 1.

**Fig. 1:**
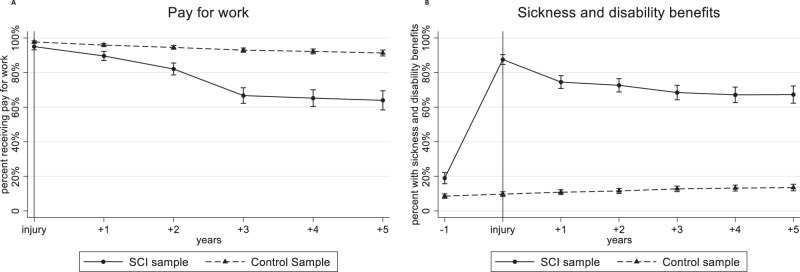
Percentages receiving (A) any amount of pay for work, and (B) sickness and disability benefits. Results of the general estimation equations’ (GEE) logistic regression models with “Receiving any amount of pay for work” (**A**, left side) and “Receiving sickness and disability benefits” (**B**, right side) as dependent variables. All models included adjustment for age, sex and educational level. Estimates from the analyses were used to calculate and graphically present the estimated percent receiving pay for work, and receiving sickness and disability benefits, with 95% confidence intervals. The vertical line at injury indicates the time of spinal cord injury. The results are shown for the SCI sample and control sample at each 1-year interval during follow-up.

The estimated percentage receiving pay for work in the SCI sample gradually decreased from 100% before injury to 63% (95% CI 57–69) six years after injury, while decline in the control sample was from 100% to 91% (95% CI 90–93) (Fig. 1A). The estimated percentage receiving sickness and disability benefits in the SCI sample was 18% before injury (95% CI 14-21), peaked to 87% during the first year after injury and then decreased to 67% (95% CI 61–72) six years after injury (Fig. 1B). In the control sample it rose from 8% before “injury” to 13% (95% CI 12–15) six years later.

**Fig. 2:**
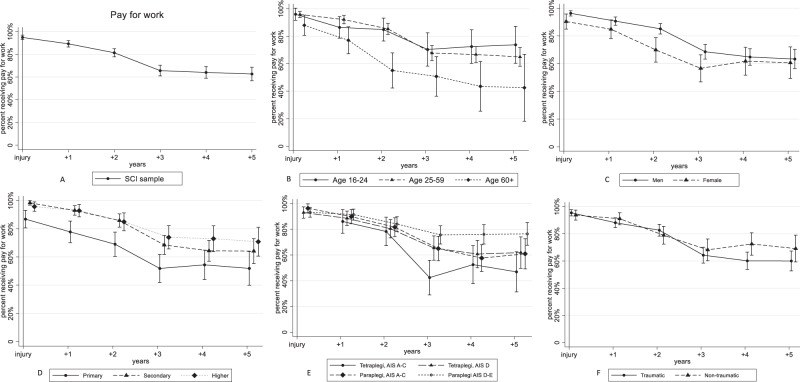
Percentages receiving “any amount of pay for work” in the SCI sample, including subgroups. Results of the general estimation equations’ (GEE) logistic regression models with “Receiving any amount of pay for work” as dependent variable. All models included adjustment for age, sex and educational level. Estimates from the analyses were used to calculate and graphically present the estimated percent receiving pay for work with 95% confidence intervals. The vertical line at injury indicates the time of spinal cord injury. AIS American Spinal Injury Association Impairment Scale. Primary, secondary and higher refer to the level of education. Traumatic refers traumatic spinal cord injury. Non-traumatic refers to non-traumatic spinal cord injury.

**Fig. 3:**
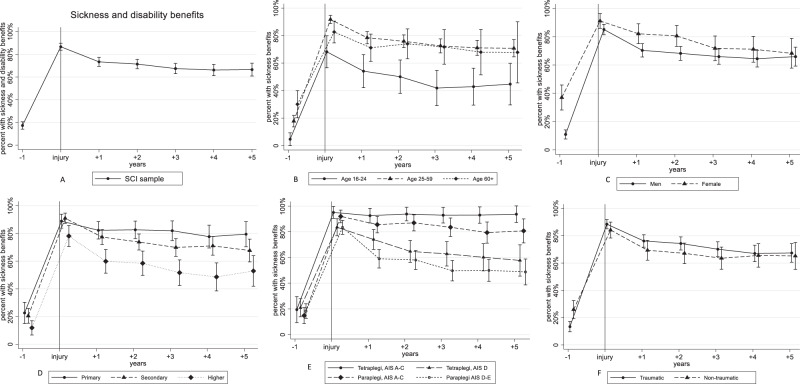
Percentages receiving “sickness and disability benefits” in the SCI sample, including subgroups. Results of the general estimation equations’ (GEE) logistic regression models with “Receiving sickness and disability benefits” as dependent variable. All models included adjustment for age, sex and educational level. Estimates from the analyses were used to calculate and graphically present the estimated percent receiving pay for work with 95% confidence intervals. The vertical line at injury indicates the time of spinal cord injury. AIS American Spinal Injury Association Impairment Scale. Primary, secondary and higher refer to the level of education. Traumatic refers traumatic spinal cord injury. Non-traumatic refers to non-traumatic spinal cord injury.

The development of labour market participation over time for the SCI sample across subgroups (age, sex, educational level, and injury characteristics) is shown in Figs. 2B–F and 3B–F.

For the SCI sample, there was a gradual shift from short-term to long-term benefits during follow-up, with over half of persons with SCI on disability pension (DP) at end of follow-up (Supplementary Fig. 1).

Among those receiving pay for work 4–6 years after SCI, 55–57 % also received sickness and disability benefits (Supplementary Table 1).

Comparison between the persons with SCI and their controls (within matched groups) showed that persons with SCI had 28%-point lower annual percentage of receiving pay for work 4–6 years after SCI, and a corresponding 36%-point higher annual percentage of receiving sickness and disability benefits (Table 3).

**Table 3:** Comparisons of spinal cord injury (SCI) patients with controls within matched groups, reporting prevalence differences (in %-points) and odds ratios (OR) with 95% confidence interval (95% CI), for receiving (1) any amount of pay for work and (2)sickness and disability benefits*.

Outcomes	Year before injury				Year 1–3 after injury				Year 4–6 after injury			
	Prevalence difference	95% CI	OR	95% CI	Prevalence difference	95% CI	OR	95% CI	Prevalence difference	95% CI	OR	95% CI
***Receiving any amount of pay for work***												
SCI vs. controls					−25%	[−30– −21%]	0.3	[0.3–0.4]	−28%	[−32– −24%]	0.1	[0.1–0.1]
***Sickness and disability benefits***												
SCI vs. controls	21%	[14– 28%]	2.4	[1.7 – 3.4]	42%	[37– 47%]	129.2	[101.1– 165.2]	36%	[31–41%]	44.3	[34.9–56.3]

*Table displays within-group estimates from fixed effect logistic regression models.

Persons with SCI with higher age at injury (age 60+), primary level of education and more severe neurological outcome (tetraplegia AIS A-C) had lower odds of labour market participation (Table 4). Compared with persons with less impairment (Paraplegia AIS D-E), persons with more severe impairment (Tetraplegia AIS A-C) had 70 % lower odds for receiving pay for work (OR 0.30, 95% CI 0.17–0.54, Model C), and about 20 times higher odds of receiving sickness and disability benefits (OR 19.6 95% CI 9.04–42.53 Model C).

**Table 4:** The impact of personal and injury characteristics on outcomes “Receiving any amount for pay for work” and “Receiving sickness and disability benefits”*.

	Receiving pay for work						Receving sickness and disability benefits					
	Model A		Model B		Model C		Model A		Model B		Model C	
	OR	95% CI	OR	95% CI	OR	95% CI	OR	95% CI	OR	95% CI	OR	95% CI
***Age group at injury***												
16–29 years	ref		ref		ref		ref		ref		ref	
30–39 years	1.22	[0.69–2.15]	0.96	[0.53–1.73]	0.81	[0.43–1.51]	2.29	[1.30–4.01]	3.48	[1.96–6.19]	3.99	[2.23–7.13]
40–49 years	0.91	[0.54–1.54]	0.73	[0.42–1.28]	0.62	[0.34–1.13]	2.64	[1.55–4.48]	4.69	[2.67–8.24]	5.67	[3.11–10.31]
50–59 years	0.73	[0.45–1.20]	0.58	[0.34–0.98]	0.40	[0.22–0.73]	3.04	[1.81–5.09]	4.94	[2.83–8.60]	6.13	[3.35–11.20]
60–66 years	0.29	[0.16–0.51]	0.25	[0.14–0.45]	0.19	[0.10–0.35]	2.00	[1.14–3.52]	2.70	[1.49–4.89]	3.23	[1.75–5.95]
***Sex***												
Female	ref		ref		ref		ref		ref		ref	
Male	1.50	[1.02–2.20]	1.67	[1.11–2.52]	1.04	[0.68–1.61]	0.61	[0.40–0.93]	0.48	[0.31–0.74]	0.73	[0.47–1.14]
***Level of education***												
Primary education	ref		ref		ref		ref		ref		ref	
Secondary education	2.18	[1.45–3.27]	2.05	[1.33–3.15]	1.47	[0.95–2.30]	0.54	[0.34–0.86]	0.52	[0.33–0.84]	0.75	[0.46–1.21]
Higher education	2.75	[1.71–4.44]	2.67	[1.62–4.39]	1.42	[0.84–2.38]	0.28	[0.16–0.46]	0.26	[0.15–0.43]	0.39	[0.23–0.65]
***SCI impairment group (level and AIS)***												
Paraplegia AIS D-E			ref		ref				ref		ref	
Tetraplegia AIS D			0.52	[0.32–0.83]	0.65	[0.40–1.04]			1.74	[1.14–2.64]	1.54	[1.02–2.33]
Paraplegia AIS A-C			0.50	[0.31–0.80]	0.50	[0.31–0.81]			5.67	[3.40–9.45]	5.45	[3.35–8.87]
Tetraplegia AIS A-C			0.29	[0.16–0.52]	0.30	[0.17–0.54]			17.92	[7.96–40.32]	19.6	[9.04–42.53]
***Cause of SCI injury***												
Traumatic SCI			ref		ref				ref		ref	
Non-traumatic SCI			1.07	[0.71–1.62]	1.45	[0.94–2.22]			1.24	[0.83–1.84]	0.85	[0.58–1.26]
***Year of injury***												
Injury 2011–2014			ref		ref				ref		ref	
Injury 2015–2017			1.00	[0.69–1.45]	0.85	[0.58–1.25]			0.88	[0.62–1.27]	1.06	[0.74–1.51]
Work income **												
0–299,999 NOK					ref						ref	
300,000–499,999 NOK					2.27	[1.41–3.65]					1.16	[0.70–1.94]
500,000–999,999 NOK					3.52	[2.06–6.00]					0.42	[0.25–0.69]
1,000,000 or above					10.69	[2.76–41.47]					0.18	[0.07–0.45]
Sickness/disability benefit***												
No benefit					ref						ref	
Benefit					0.36	[0.24–0.53]					3.31	[2.19–5.00]
Having an employer ***												
No					ref						ref	
Yes					1.38	[0.84–2.26]					0.86	[0.49–1.49]

*Table displays results of the logistic regression models (GEE models with repeated annual observations per individual).

Model A Adjusted for time, age groups, sex, level of education.

Model B Adjusted for time, age groups, sex, level of education, SCI impairment groups, cause of injury and year of injury.

Model C Adjusted for time, age groups, sex, level of education, SCI impairment groups, cause of injury and year of injury. employment income before injury, employment status before injury and medical benefits before injury.

**During year before injury.

***In at least one month in year before injury.

*OR* Odds Ratio, *CI* Confidence Interval, *NOK* Norwegian kroner, *EUR* Euro, *SCI* spinal cord injury, *AIS* American Spinal Injury Association Impairment Scale.

Differences in pre-injury employment (employment income, employment status and receipt of benefits) had a major influence on outcomes of labour participation after injury (Table 4).

The predicted probabilities for labour participation are shown in supplementary Table 2.

Results of the supplementary analysis are shown in Supplementary Fig. 1, Supplementary Fig. 2 and Supplementary Table 3.

## Discussion

### Level of labour market participation post-spinal cord injury

4-6 Years after injury, the percentage receiving pay for work among persons with SCI was 28%-points lower and the percentage receiving sickness and disability benefits 36%-point higher, compared with their matched controls from the general population. Thus, as expected, this study confirms the common notion and earlier studies showing that a SCI injury reduced labour market participation [2].

Our finding that 63% received pay for work six years post-injury is higher than the level reported from most other studies from Norway (35% to 52%, [4, 5, 7, 12]) and internationally (10% to 61% [7]). However, these studies have used different definitions of work activity, preventing a direct comparison, a problem that has been reported in literature reviews [2, 13, 22]. A recently published systematic review showed that 54 % of the studies used a salary-dependent definition such as “working for pay” or “earning minimum wage” [13]. The definition used in our study, being registered in the tax system as receiving any pay, was inclusive and led to a higher level than e.g., a definition of including those earning more than the minimum wage would have given. Despite the relatively high proportion receiving pay for work in this study, the considerably lower mean income levels compared with the matched controls indicate that many people who were employed after SCI still may not have achieved a satisfactory level of employment.

When it comes to the level receiving sickness and disability benefits, which was 67% after 6 years post-injury in our study, there are similar challenges regarding direct comparison. For previous studies with linkages to national registries, the levels reported have been 41 % 5 years after severe trauma in Norway [23], and 24% 5 years after mild traumatic brain injury in Denmark [24]. In our study, the high post-injury level of benefit receipt was probably partly influenced by the pre-injury level of sickness and disability benefits in the SCI sample (17%), which was elevated compared to controls (9%). This difference in pre-injury benefit status is in line with findings from a nationwide Danish register-based SCI study that showed approximately two times higher health care costs for persons with SCI two years before injury, compared to controls [25]. We found their explanation, that this might be related to ongoing disease in advance of non-traumatic SCI, and a traumatic SCI group that could be more accident-prone, both resulting in higher costs, plausible and relevant for our findings.

### Increasing labour market participation

Even if the current study showed a relatively high level of labour market participation for persons with SCI, efforts should be made to increase it further, especially because a considerable group is not included in the labour market after SCI (mainly the more severely injured and low educated persons). At the same time, the importance of work in people’s lives is well known [1, 26].

The factors found in this study to influence labour market participation following SCI, neurological outcome, level of education, age at injury, time since injury, and pre-injury attachment to employment, strongly confirming findings from previous studies [2–6]. Of these, gaining additional education is a factor that can be modified after the injury [3]. Higher educated persons are more often in non-physically demanding employment compared to those with lower education [27]. Consequently, persons with higher education post-SCI tend to have more career opportunities open to them [27]. Thus, promoting vocational re-training towards jobs requiring higher education is likely to be a valid approach to increase the level of labour market participation [27–29].

But also measures taken at the workplace should be considered. One example is assistive technologies which have been suggested to be helpful for those with limited cognitive resources to do physically oriented jobs [27]. Also increased employer incentives, such as obligations regarding offering a suitable job and providing workplace adaptations could be useful [9]. Previous research has indicated that the role of the employers is underutilised in Norway [5].

### Study strengths and limitations

Strength of this study is the nationwide register-based and controlled design, with clinical patient data from a national medical SCI quality registry linked with complete individual national registry data on employment, education, income, and social security benefits. This linkage provides high accuracy and quality owing to the use of the unique identity number assigned to all Norwegians. Use of registry data minimizes the risk of information bias, compared with patient-reported outcome measures [30]. Moreover, we had access to data of the general population and could therefore provide a detailed comparison between people with SCI and the general population.

There are some noteworthy limitations. In general, one should be careful with making causal interpretations of the findings. Registry data provide limited information on personal characteristics (including health status) to be used as adjustment variables in the regression analyses. In addition, registry data is not well suited to study quality of employment that would be of importance for labour market participation after SCI, such as the promotions and job satisfaction.

Only persons with SCI that received pay for work in the year before injury were included in this study, which means that the results are not generalizable to all persons with SCI. Furthermore, the number of control persons per patient varied from zero to five, introducing some imbalance in the composition of the control sample. The comparisons between the SCI sample and control sample must therefore be interpreted with caution. However, we have taken this imbalance into account by performing analyses within matched groups.

The outcome pay for work is based on annual registrations, with the consequence that those with SCI are registered with income the year of their injury.

## Conclusion

Labour market participation clearly decreased after injury among persons with SCI. Even though a relatively large proportion of those who participated in the labour market before SCI still did so after injury, more than half of these also depended on sickness and disability benefits. Even stronger emphasis of vocational re-training towards jobs requiring higher education, more flexible workplace adaptations and more employer incentives should be considered to increase labour market participation for persons with SCI.

## Supplementary information

Supplementary table 1

Supplementary table 2

Supplementary table 3

Supplementary figure 1

Supplementary figure 2

Supplementary figure legend

## Data Availability

The data used in this study are from the NorSCIR, SSB and NAV. There are restrictions on the use of data from national registries. These data were used under licence for the current study and are not publicly available.
