# Supplementary material for: Labour market participation after spinal cord injury. A register-based cohort study
Source: Spinal Cord. 2023 Jan 30;61(4):244–52. doi: 10.1038/s41393-023-00876-4 (PMC10070183; doi:10.1038/s41393-023-00876-4)
Supplement: Supplementary file 1 — Supplementary table 1 [file 41393_2023_876_MOESM1_ESM.docx]

**Supplementary table 1. The number and percentage of participants in the spinal cord injury (SCI) sample and control sample**

**who received (+) or did not receive (-) pay for work and/or sickness and disability benefits each year of follow-up.**

|  |  | Year of follow-up (before/after injury) | | | | | | |
| --- | --- | --- | --- | --- | --- | --- | --- | --- |
|  |  | -1-0 | 0-1 | 1-2 | 2-3 | 3-4 | 4-5 | 5-6 |
| N included SCI sample | | 451 | 451 | 436 | 414 | 345 | 283 | 202 |
|  |  |  |  |  |  |  |  |  |
|  | Receiving any amount of pay for work +  Sickness and disability benefits - | 374 (83%) | 55 (12%) | 111 (25%) | 115 (28%) | 100 (29%) | 84 (30%) | 60 (30%) |
|  | Receiving any amount of pay for work +  Sickness and disability benefits + | 77 (17%) | 372 (82%) | 279 (64%) | 225 (54%) | 133 (39%) | 105 (37%) | 74 (37%) |
|  | Receiving any amount of pay for work -  Sickness and disability benefits - |  | 5 (1%) | 5 (1%) | 4 (1%) | 7 (2%) | 6 (2%) | 5 (2%) |
|  | Receiving any amount of pay for work -  Sickness and disability benefits + |  | 19 (4%) | 41 (9%) | 70 (18%) | 105 (30%) | 88 (31%) | 63 (32%) |
|  |  |  |  |  |  |  |  |  |
| N included control sample | | 1791 | 1790 | 1742 | 1700 | 1444 | 1192 | 841 |
|  |  |  |  |  |  |  |  |  |
|  | Receiving any amount of pay for work +  Sickness and disability benefits - | 1637 (91%) | 1588 (89%) | 1522 (87%) | 1467 (86%) | 1226 (85%) | 1010 (85%) | 715 (85%) |
|  | Receiving any amount of pay for work +  Sickness and disability benefits + | 154 (9%) | 157 (9%) | 147 (8%) | 143 (8%) | 124 (9%) | 99 (8%) | 63 (8%) |
|  | Receiving any amount of pay for work -  Sickness and disability benefits - |  | 27 (2%) | 34 (2%) | 41 (2%) | 40 (3%) | 37 (3%) | 23 (3%) |
|  | Receiving any amount of pay for work -  Sickness and disability benefits + |  | 18 (1%) | 39 (2%) | 49 (3%) | 54 (4%) | 49 (4%) | 35 (4%) |
