# Supplementary material for: Labour market participation after spinal cord injury. A register-based cohort study
Source: Spinal Cord. 2023 Jan 30;61(4):244–52. doi: 10.1038/s41393-023-00876-4 (PMC10070183; doi:10.1038/s41393-023-00876-4)
Supplement: Supplementary file 3 — Supplementary table 3 [file 41393_2023_876_MOESM3_ESM.docx]

**Supplementary table 3. The distribution of participants in the spinal cord injury sample (SCI) and control sample in employment income groups each year of follow-up. N (%).**

|  |  | Year of follow-up (before/after injury) | | | | | | |
| --- | --- | --- | --- | --- | --- | --- | --- | --- |
|  |  | -1-0 | 0-1 | 1-2 | 2-3 | 3-4 | 4-5 | 5-6 |
| N included SCI sample | | 451 | 451 | 436 | 414 | 345 | 283 | 202 |
|  | 0 or less NOK | 0 (0%) | 24 (5%) | 46 (11%) | 74 (18%) | 112 (32%) | 94 (33%) | 68 (34%) |
|  | 0-299.999 NOK | 171 (38%) | 172 (38%) | 263 (60%) | 226 (55%) | 135 (39%) | 112 (40%) | 77 (38%) |
|  | 300.000 -499.999 NOK | 130 (29%) | 141 (31%) | 54 (12%) | 52 (13%) | 36 (10%) | 27 (10%) | 22 (11%) |
|  | 500.000-999.999 NOK | 129 (29%) | 96 (21%) | 60 (14%) | 48 (12%) | 54 (16%) | 46 (16%) | 34 (17%) |
|  | More than 1.000.000 NOK | 21 (5%) | 18 (4%) | 13 (3%) | 14 (3%) | 8 (2%) | 4 (1%) | 1 (1%) |
|  |  |  |  |  |  |  |  |  |
| N included control sample | | 1780 | 1777 | 1732 | 1687 | 1432 | 1184 | 837 |
|  | 0 or less NOK | 0 (0%) | 45 (3%) | 73 (4%) | 90 (5%) | 94 (7%) | 83 (7%) | 58 (7%) |
|  | 0-299.999 NOK | 598 (34%) | 552 (31%) | 488 (28%) | 410 (24%) | 304 (21%) | 229 (19%) | 153 (18%) |
|  | 300.000 -499.999 NOK | 589 (33%) | 550 (31%) | 508 (29%) | 491 (29%) | 392 (27%) | 317 (27%) | 213 (25%) |
|  | 500.000-999.999 NOK | 536 (30%) | 566 (32%) | 589 (34%) | 619 (37%) | 566 (40%) | 489 (41%) | 364 (43%) |
|  | More than 1.000.000 NOK | 57 (3%) | 64 (4%) | 74 (4%) | 77 (5%) | 76 (5%) | 66 (6%) | 49 (6%) |

Abbreviation: NOK, Norwegian kroner.
