# Supplementary figures and images for: Labour market participation after spinal cord injury. A register-based cohort study
Source: Spinal Cord. 2023 Jan 30;61(4):244–52. doi: 10.1038/s41393-023-00876-4 (PMC10070183; doi:10.1038/s41393-023-00876-4)

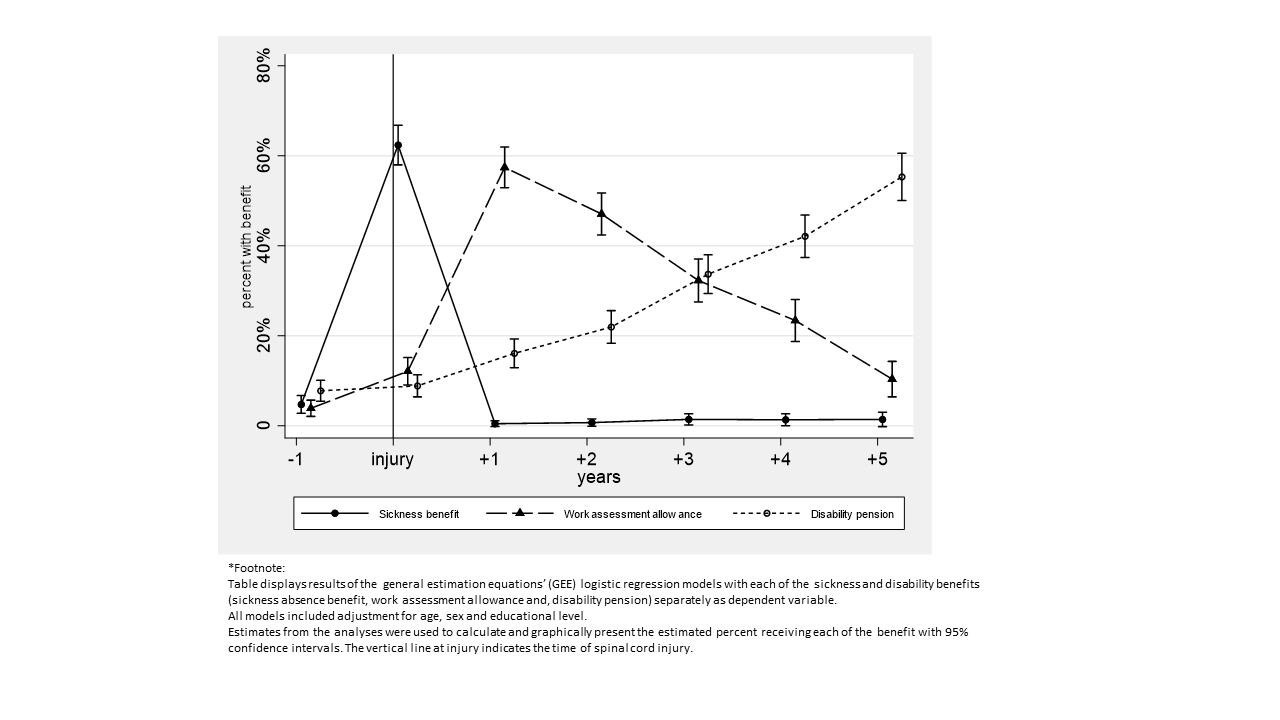

Supplement: Supplementary file 4 — Supplementary figure 1 [file 41393_2023_876_MOESM4_ESM.tif]

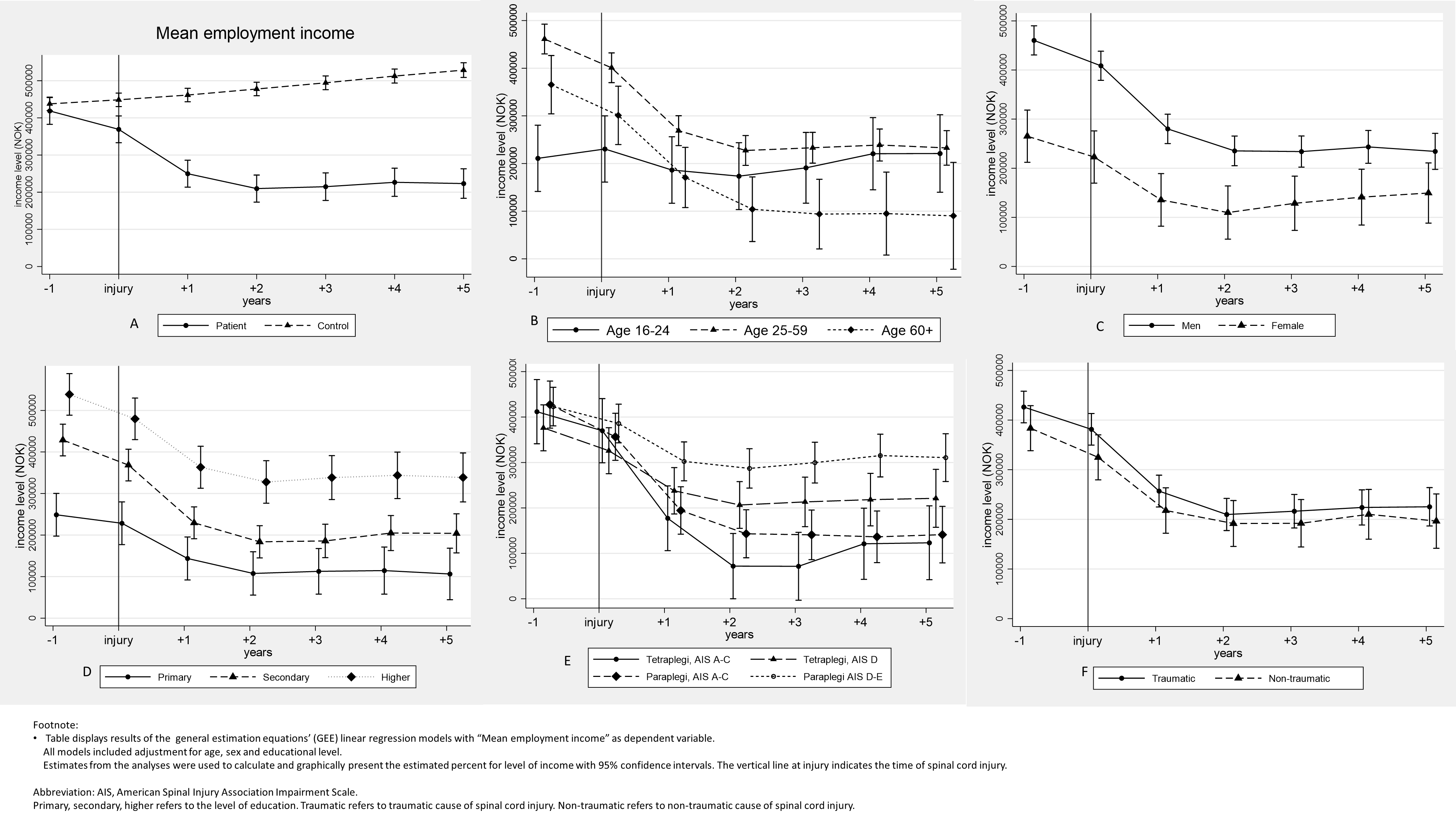

Supplement: Supplementary file 5 — Supplementary figure 2 [file 41393_2023_876_MOESM5_ESM.tif]
