# Supplementary material for: Labour market participation after spinal cord injury. A register-based cohort study
Source: Spinal Cord. 2023 Jan 30;61(4):244–52. doi: 10.1038/s41393-023-00876-4 (PMC10070183; doi:10.1038/s41393-023-00876-4)
Supplement: Supplementary file 6 — Supplementary figure legend [file 41393_2023_876_MOESM6_ESM.docx]

Supplementary figure 1. Percentage of persons with spinal cord injury receiving different types of sickness and disability benefits over time during follow-up*.

Supplementary figure 2. Mean level of employment income at each 1-year interval during follow-up*. (Figures B, C, D, E, F for individuals with spinal cord injury only.)
